# Active and passive case-finding in tuberculosis-affected households in Peru: a 10-year prospective cohort study

**DOI:** 10.1016/S1473-3099(18)30753-9

**Published:** 2019-05

**Authors:** Matthew J Saunders, Marco A Tovar, Dami Collier, Matthew R Baldwin, Rosario Montoya, Teresa R Valencia, Robert H Gilman, Carlton A Evans

**Affiliations:** a Infectious Diseases and Immunity, Imperial College London, and Wellcome Trust Imperial College Centre for Global Health Research, London, UK; b Innovation for Health and Development (IFHAD), Laboratory of Research and Development, Universidad Peruana Cayetano Heredia, Lima, Peru; c Innovación Por la Salud Y Desarrollo (IPSYD), Asociación Benéfica PRISMA, Lima, Peru; d Columbia University, College of Physicians and Surgeons, New York, NY, USA; e Johns Hopkins Bloomberg School of Public Health, Baltimore, MD, USA

## Abstract

**Background:**

Active case-finding among contacts of patients with tuberculosis is a global health priority, but the effects of active versus passive case-finding are poorly characterised. We assessed the contribution of active versus passive case-finding to tuberculosis detection among contacts and compared sex and disease characteristics between contacts diagnosed through these strategies.

**Methods:**

In shanty towns in Callao, Peru, we identified index patients with tuberculosis and followed up contacts aged 15 years or older for tuberculosis. All patients and contacts were offered free programmatic active case-finding entailing sputum smear microscopy and clinical assessment. Additionally, all contacts were offered intensified active case-finding with sputum smear and culture testing monthly for 6 months and then once every 4 years. Passive case-finding at local health facilities was ongoing throughout follow-up.

**Findings:**

Between Oct 23, 2002, and May 26, 2006, we identified 2666 contacts, who were followed up until March 1, 2016. Median follow-up was 10·0 years (IQR 7·5–11·0). 232 (9%) of 2666 contacts were diagnosed with tuberculosis. The 2-year cumulative risk of tuberculosis was 4·6% (95% CI 3·5–5·5), and overall incidence was 0·98 cases (95% CI 0·86–1·10) per 100 person-years. 53 (23%) of 232 contacts with tuberculosis were diagnosed through active case-finding and 179 (77%) were identified through passive case-finding. During the first 6 months of the study, 23 (45%) of 51 contacts were diagnosed through active case-finding and 28 (55%) were identified through passive case-finding. Contacts diagnosed through active versus passive case-finding were more frequently female (36 [68%] of 53 *vs* 85 [47%] of 179; p=0·009), had a symptom duration of less than 15 days (nine [25%] of 36 *vs* ten [8%] of 127; p=0·03), and were more likely to be sputum smear-negative (33 [62%] of 53 *vs* 62 [35%] of 179; p=0·0003).

**Interpretation:**

Although active case-finding made an important contribution to tuberculosis detection among contacts, passive case-finding detected most of the tuberculosis burden. Compared with passive case-finding, active case-finding was equitable, helped to diagnose tuberculosis earlier and usually before a positive result on sputum smear microscopy, and showed a high burden of undetected tuberculosis among women.

**Funding:**

Wellcome Trust, Department for International Development Civil Society Challenge Fund, Joint Global Health Trials consortium, Bill & Melinda Gates Foundation, Imperial College National Institutes of Health Research Biomedical Research Centre, Foundation for Innovative New Diagnostics, Sir Halley Stewart Trust, WHO, TB REACH, and IFHAD: Innovation for Health and Development.

## Introduction

Reducing tuberculosis transmission in households and communities entails early diagnosis of people living with tuberculosis followed by rapid initiation of appropriate treatment before tuberculosis becomes sputum smear-positive and more infectious.^1^ WHO estimates that, in 2017, more than a third of people living with tuberculosis were neither diagnosed, treated, nor reported, representing an enormous barrier to tuberculosis control and elimination.^2^ In most countries of low and middle income with a high burden of tuberculosis, investigation for tuberculosis usually only starts when people present to health services with symptoms suggestive of pulmonary tuberculosis, termed passive case-finding. WHO recommends that contacts of newly diagnosed patients are systematically screened because of their well-established high risk of disease.^3^, ^4^ However, this active case-finding among contacts—termed contact investigation—is inconsistently implemented in settings with a high burden of tuberculosis, and evidence to inform its optimum delivery is scant.^5^

Most tuberculosis cases and deaths are reported in men.^2^ Analyses of prevalence surveys consolidate these case-notification data and show that overall tuberculosis prevalence is twice as high in men than in women.^6^ However, these data are mostly from southeast Asia, Africa, and the western Pacific, with only two small subnational surveys in relatively isolated indigenous communities from Latin America contributing to the analysis. In Latin America, case notifications are consistently higher in men and, in 2015, 61% of cases notified in Peru were reported in men.^2^, ^7^ However, prevalence surveys of indigenous communities from Ecuador^8^ and Brazil^9^ imply a more equal tuberculosis prevalence by sex. In previous work in Peru, we found tuberculosis diagnostic delay to be greater among women than men,^10^ and qualitative research showed a common perception that women's health and tuberculosis care was of secondary importance to that of men, hampering equitable access to health care.^11^ These observations support the hypothesis that the observed sex difference in tuberculosis notifications in Peru might be partly accounted for by underdetection of tuberculosis in women.

**Research in context**

**Evidence before this study**

We searched PubMed, Google Scholar, Web of Science, and Embase for studies published up to Oct 1, 2018, that investigated active case-finding among contacts of patients with tuberculosis. Search terms included, but were not restricted to, “tuberculosis”, “contact tracing”, “contact investigation”, “active case-finding”, “sex”, “prevalence”, and “passive case-finding”. We did not restrict this search by language. This search retrieved several observational studies describing active case-finding interventions and identified a large randomised controlled trial that showed the effectiveness of active case-finding for increasing case detection among contacts in a high-prevalence setting. However, few data described the relative contribution to the overall tuberculosis case burden of active versus passive case-finding among contacts over a prolonged period. Although active case-finding is generally considered to be important for early case detection, our search showed little evidence comparing disease and demographic characteristics between individuals diagnosed through active versus passive case-finding. We also searched PubMed, Google Scholar, Web of Science, and Embase for studies investigating the relation between tuberculosis and sex. Search terms included, but were not restricted to, “tuberculosis”, “contact tracing”, “contact investigation”, “active case-finding”, “sex”, “prevalence”, and “passive case-finding”. We did not restrict this search by language. This search retrieved a systematic review characterising sex differences in tuberculosis burden in countries of low and middle income. A significantly higher prevalence of tuberculosis was reported in men, and findings suggested that men are disadvantaged in accessing tuberculosis care in many settings. In Latin America, although case notifications are higher in men, two small prevalence surveys have shown a similar tuberculosis burden among men and women. Furthermore, our research group—IFHAD (Innovation For Health And Development)—has previously described a longer tuberculosis diagnostic delay among women and a perception that in our setting, Peru, women's health is generally perceived to have lower priority than men's, acting as a barrier to accessing health care. Taken together, these observations suggest that there could be a large burden of undiagnosed tuberculosis in women in our setting.

**Added value of this study**

In our prospective cohort study, we showed a very high risk for tuberculosis that persisted for at least 10 years among contacts aged 15 years or older of patients with laboratory-confirmed tuberculosis. Active case-finding made an important contribution to tuberculosis diagnoses in this group, and intensified active case-finding with repeat household visits and sputum culture testing detected more cases than did programmatic active case-finding, which relied on contacts to visit health posts for sputum smear microscopy. Active case-finding was equitable and, compared with passive case-finding, detected tuberculosis earlier and when contacts were less likely to have highly infectious sputum smear-positive tuberculosis. Importantly, active case-finding detected a large burden of undetected tuberculosis among women, reversing the apparent sex difference in tuberculosis case notifications. Despite the important contribution active case-finding made to tuberculosis diagnoses, during 10 years of follow up, passive case-finding was used to identify most of the tuberculosis case burden among contacts.

**Implications of all the available evidence**

Adult contacts of patients with tuberculosis should be a priority group for tuberculosis screening, preventive treatment, and long-term surveillance. Active case-finding interventions can make an important contribution to diagnosing tuberculosis among these contacts and might be improved by more intensive frequent testing, using more sensitive and rapid diagnostics and more relaxed symptomatic criteria for screening. However, such interventions should also be integrated with initiatives to concurrently improve access to, and quality of, passive case-finding. In our setting, active case-finding was equitable, detected tuberculosis at an earlier and less infectious stage, and showed a high burden of undetected tuberculosis in women, suggesting the apparent sex differences in tuberculosis case notifications in Peru might, at least in part, be accounted for by underdetection of tuberculosis in women.

In this study, we investigated the long-term burden of tuberculosis in contacts of patients with tuberculosis, described the relative contribution of active and passive case-finding to tuberculosis case detection, and compared sex and disease characteristics between contacts diagnosed through active versus passive case-finding. These data characterise the epidemiology of tuberculosis among contacts in Peru, informing the optimum delivery of active case-finding among contacts that might be applicable in other countries of low and middle income, and provide important insights into the nature of sex differences in tuberculosis burden.

## Methods

### Participants

We did a prospective cohort study of contacts of index patients with tuberculosis from 16 desert shanty towns in Ventanilla, Callao, Peru. The study setting is fully described elsewhere in a detailed analysis of risk factors for secondary tuberculosis among the same contacts included in this study.^4^

Index patients were defined as patients registered to receive treatment in Peruvian Ministry of Health (MINSA)-run health posts who had laboratory-confirmed pulmonary tuberculosis, which in this setting almost always implied a positive result on sputum smear microscopy. Index patients were the first individual we identified with tuberculosis among household members and they were eligible for inclusion in this study if they had at least one contact aged 15 years or older. They were not invited to participate if they had been previously recruited or were a contact of a patient whom we had already recruited, so households were only invited to participate once.

Contacts were defined as individuals aged 15 years or older who reported being in the same house as the index patient for more than 6 h per week in the 2 weeks preceding the index patient's diagnosis. Contacts were ineligible if they were taking or awaiting tuberculosis treatment at the time of recruitment. During the study period, contacts aged 15 years or older were not routinely offered isoniazid preventive treatment, in accordance with national policy at the time.^12^ We restricted our investigation to contacts aged 15 years or older because, in this setting, younger contacts might receive preventive treatment and are often treated for tuberculosis empirically, confounding interpretation of tuberculosis diagnosis in children.

We obtained written informed assent or consent for all recruited contacts and index patients. Ethics approval for the study was obtained from the Callao Ministry of Health (Peru), the Asociación Benéfica PRISMA (Peru), and Imperial College London (UK). The study was done with the approval and collaboration of the Peruvian national tuberculosis programme.

### Procedures

Study research nurses worked in collaboration with health posts to recruit index patients when they were diagnosed with tuberculosis. Index patients were invited to give a sputum sample, which was tested by smear microscopy and the microscopic-observation drug-susceptibility (MODS) assay including liquid and solid Lowenstein-Jensen culture.^13^ Study nurses then visited the index patient's household and completed a census of all contacts. Contacts were invited to participate in a randomised controlled trial of micronutrient supplementation to prevent tuberculosis.^14^ Because micronutrient supplementation was shown not to affect tuberculosis rates,^14^ contacts are included in our study irrespective of allocation. All index patients and contacts recruited completed a baseline questionnaire characterising demographics, socioeconomic factors, and other tuberculosis risk factors.^4^

All contacts were followed up for tuberculosis diagnosis by checking national tuberculosis programme treatment registers and cards in participating MINSA-run health posts. On diagnosis, and at subsequent household visits, we characterised tuberculosis episodes among contacts, including asking about symptom duration, and we asked about both extrapulmonary and pulmonary tuberculosis diagnosed and treated outside the jurisdiction of the health posts in Ventanilla. Self-reported tuberculosis episodes were confirmed against treatment registers in participating MINSA-run health posts whenever possible.

### Outcomes

The primary outcome of our cohort study was tuberculosis diagnosis among contacts. Time to tuberculosis was defined from the date the index patient initiated tuberculosis treatment until the date the contact was diagnosed with tuberculosis, or if this date was unavailable, the date they initiated treatment. For contacts not diagnosed with tuberculosis, follow-up was censored at the date they were last known to be alive and free of tuberculosis. All participating contacts underwent integrated programmatic active case-finding, intensified study active case-finding, and programmatic passive case-finding. Any case of tuberculosis ascertained through one of these three strategies was considered an incident case in the follow-up period.

### Programmatic active case-finding

During the study, MINSA recommended active case-finding for contacts, which principally entailed clinical assessment and sputum smear microscopy without culture testing. Peruvian national guidelines recommended collecting two spot samples from contacts with cough for longer than 2 weeks, although in practice one or two sputum samples were typically obtained from all contacts, irrespective of symptoms.^12^ Chest radiography was not recommended and only rarely done for contacts at the discretion of the assessing doctor. All contacts were eligible for this programmatic active case-finding, which (except for chest radiography) was provided free of direct charges at participating MINSA-run health posts. This programmatic active case-finding was not influenced by our study.

### Intensified study active case-finding

Study research nurses visited households at recruitment then again after 2 weeks, 4 weeks, 6 weeks, and 8 weeks, and then every 4 weeks throughout the index patient's treatment (usually for 6 months but could be longer for patients with multidrug-resistant tuberculosis). During these visits, study nurses offered active case-finding to all participating contacts. This entailed offering free sputum testing for contacts who had any symptom suggestive of tuberculosis—ie, cough of any duration and type (whether productive or not), fever, night sweats, weight loss, or chest pain. Spot sputum samples were obtained with careful instruction in the privacy of the contact's own home.^15^ The sample provided (whether it appeared macroscopically to be sputum or saliva) was tested by smear microscopy and cultured using the MODS assay. After completion of the index patient's treatment, we visited households on three occasions, approximately every 4 years. At these visits, we offered all available contacts free sputum testing with smear microscopy and culture using the thin-layer agar MDR/XDR-TB Colour Test.^16^ At the first two visits, testing was offered only to contacts who had symptoms. At the final visit, testing was offered to all contacts. If a contact was not present at the time of an active case-finding visit, another household member completed the screening questions on behalf of the absent contact and sputum pots were left with instructions and collected 24 h later.

### Programmatic passive case-finding

Throughout the follow-up period, contacts had access to passive case-finding at MINSA-run health posts, free of direct charges, both inside and outside the study area. The main diagnostic test available for tuberculosis during the study period was (and remains) smear microscopy of sputum, usually obtained with little instruction. Chest radiography was done at the contact's expense and was interpreted by the consulting doctor and rarely by a radiologist. Investigation for tuberculosis was typically initiated among individuals presenting to health posts with cough for longer than 2 weeks.^12^ Tuberculosis in this setting was also diagnosed and treated in MINSA-run hospitals, employer-insured health facilities for people with formal employment, prisons, and private clinics.

### Statistical analysis

Continuous data were examined for normality and summarised by medians and IQRs because they were skewed. Continuous data were compared using the Mann-Whitney U test. Categorical data were summarised as proportions and compared using the two-sample proportion test. Because missing data were infrequent, occurring in fewer than 5% of cases for baseline characteristics, the median value was used to complete baseline data. For every year after initiation of the index patients' treatment, we calculated tuberculosis incidence per 100 person-years and generated 95% CIs based on the Poisson distribution. We plotted these data against community tuberculosis incidence, which was calculated using the average tuberculosis case notification rate among adults aged 15 years or older in Ventanilla between 2002 and 2014, adjusted by 20% to correct for underdiagnosis and reporting because this value is the estimated case detection gap in Peru.^7^ We compared tuberculosis incidence between male and female contacts by calculating incidence rate ratios (IRRs) using the Stata command *stir*. We derived and plotted Kaplan-Meier curves to calculate cumulative tuberculosis risk over time.

The 6-monthly rolling average and cumulative proportion of contacts diagnosed with tuberculosis were plotted by tuberculosis ascertainment strategy and compared overall and at the end of 6 months because this time was when both programmatic and intensified study active case-finding were principally implemented. To elucidate sex differences in tuberculosis diagnosis by ascertainment strategy, we compared the proportion of people who were female between contacts diagnosed through both programmatic and intensified study active case-finding (referred to collectively as all active case-finding), contacts diagnosed through programmatic passive case-finding, index patients (who had been principally diagnosed through programmatic passive case-finding), the national proportion of new cases notified in 2015, and regional and global incidence data.^2^, ^7^ We compared overall symptom duration before diagnosis between contacts diagnosed through all active case-finding versus programmatic passive case-finding for contacts with data available and plotted the proportion of contacts who had symptoms for fewer than 15 days, 30 days, 60 days, and 90 days, to show differences at relevant thresholds of symptom duration. Finally, we compared sputum smear grade and treatment success (defined as cured or completed treatment, according to national treatment registers) proportions between contacts diagnosed through all active case-finding versus programmatic passive case-finding. To assess the socioeconomic equity of active case-finding, we compared household secondary education and household income between contacts diagnosed through all active case-finding versus programmatic passive case-finding.

All analyses were done using Stata (version 13). p values were two-sided with significance assessed at the 5% level.

### Role of the funding source

The funders had no role in study design, data collection, data analysis, data interpretation, or writing of the report. The corresponding author had full access to all the data in the study and had final responsibility for the decision to submit for publication.

## Results

**Figure 1:**
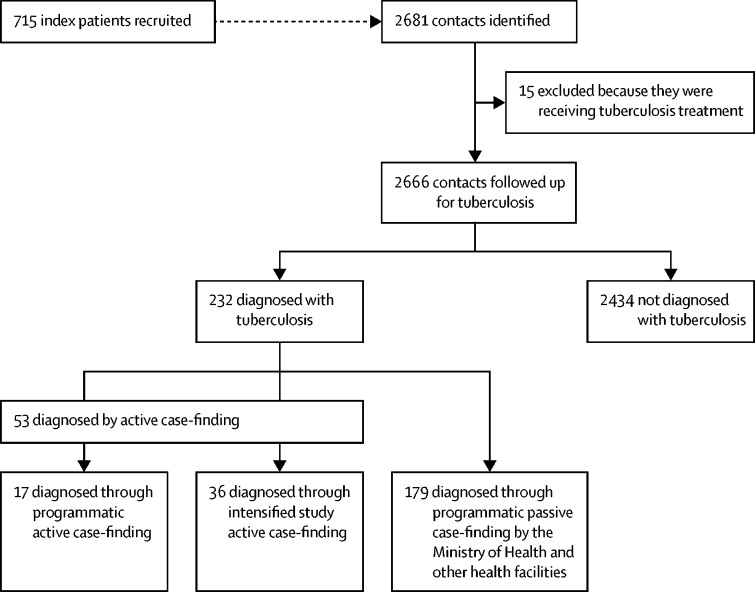
Study profile

Between Oct 23, 2002, and May 26, 2006, 715 index patients were diagnosed with tuberculosis through programmatic passive case-finding and were recruited (figure 1). The median age of index patients was 27 years (IQR 20–36). 290 (41%) index patients were female (table). 2681 contacts of index patients were assessed for study eligibility, of whom 15 (ten males and five females) were excluded because they were taking or awaiting tuberculosis treatment. The study population therefore included 2666 contacts. The median age of contacts at recruitment was 29 years (IQR 21–42). 1429 (54%) contacts were female and 1237 (46%) were male. Contacts were followed up until March 1, 2016. Follow-up was for a total of 23 758 person-years, with female contacts followed up for 13 047 person-years and male contacts for 10 711 person-years. Median follow up was 10·0 years per contact (IQR 7·5–11·0). During follow-up, 80 (3%) contacts died.

**Table:** Baseline characteristics of contacts, index patients, and households

		**Total**
**Contacts (n=2666)**		
Age at recruitment (years)		29 (21–42)
Male		1237 (46%)
Female		1429 (54%)
Known to have HIV infection		4 (<1%)
**Index patients (n=715)**		
Age at recruitment (years)		27 (20–36)
Male		425 (59%)
Female		290 (41%)
Sputum smear status		
	Negative	16 (2%)
	Positive, grade 1	253 (35%)
	Positive, grade 2	210 (29%)
	Positive, grade 3	236 (33%)
Drug susceptibility		
	Sensitive	581 (81%)
	Isoniazid monoresistant	59 (8%)
	Multidrug resistant*	75 (11%)
**Household (n=715)**		
Principally cook with kerosene or solid cooking fuels (wood, coal, animal dung, or crop wastes)		258 (36%)
Wall material		
	Adobe	77 (11%)
	Wood	344 (48%)
	Cement or brick	294 (41%)
Floor material		
	Dirt	258 (36%)
	Cement	415 (58%)
	Tiles or laminated	42 (6%)
Access to piped water inside the house		327 (46%)
Access to a toilet inside the house		297 (42%)
Electric lighting		662 (93%)
Asset ownership		
	Television	649 (91%)
	Stove	696 (97%)
	Fridge	319 (45%)
Head of household did not complete secondary education		429 (60%)
Rooms in the household		3 (2–3)
People sleeping in the household		5 (4–6)
Crowding (more than two people sleeping per room)		268 (37%)
Monthly household income (US$)†		153 (98–214)

Data are median (IQR) or n (%). Missing data were imputed using the median value because no variables had more than 5% of data missing.

* Multidrug-resistant tuberculosis was defined in patients initially prescribed a multidrug-resistant tuberculosis treatment regimen or who had microbiological evidence of resistance to rifampicin and isoniazid.

† Data were obtained in Peruvian Soles (PEN) and converted to US$ using the exchange rate US$1=PEN3·27 (July 13, 2018).

**Figure 2:**
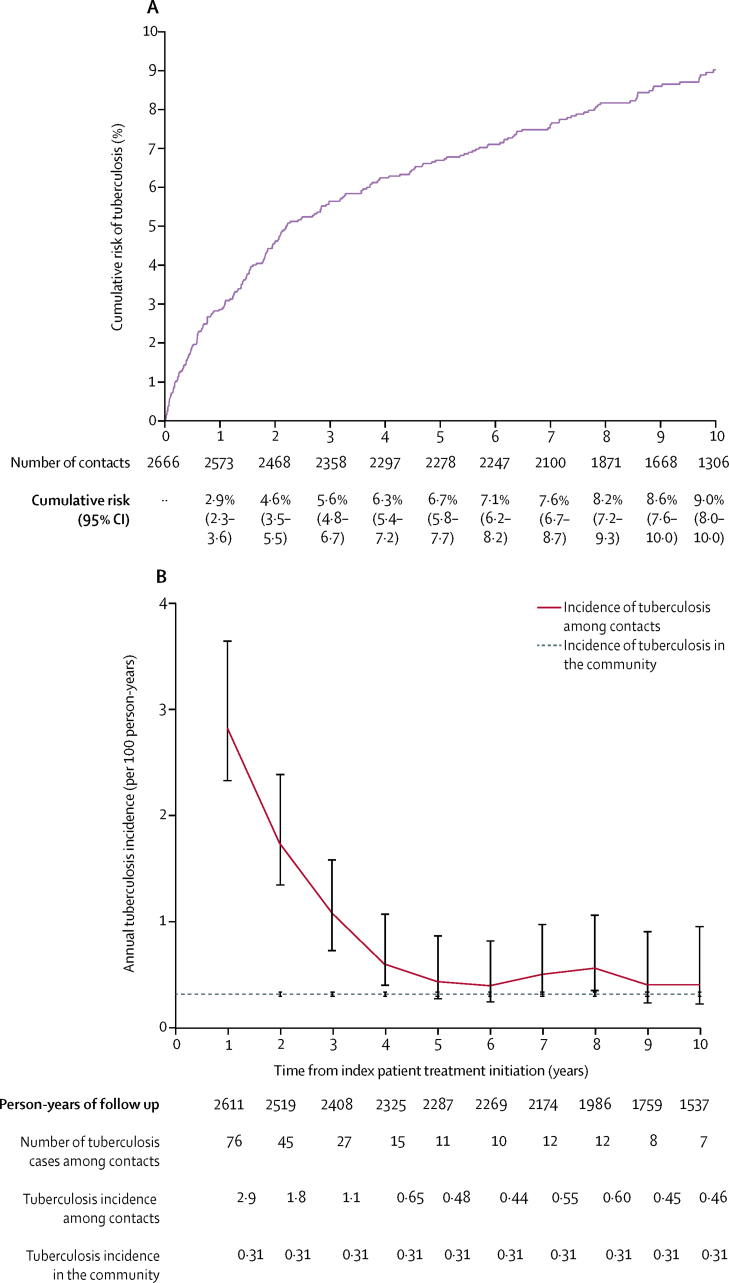
Cumulative risk and incidence of tuberculosis among contacts Error bars represent 95% CIs.

232 (9%) contacts were diagnosed with tuberculosis during the follow-up period, of whom 121 (52%) were female and 111 (48%) male. The 2-year cumulative risk of tuberculosis was 4·6% (95% CI 3·5–5·5; figure 2A). The overall incidence of tuberculosis was 0·98 cases (95% CI 0·86–1·10) per 100 person-years. Incidence of tuberculosis in female contacts was 0·93 cases per 100 person-years and in male contacts was 1·00 cases per 100 person-years. Tuberculosis incidence did not differ between male and female contacts (IRR 1·10, 95% CI 0·86–1·50; p=0·4; appendix). Overall incidence of tuberculosis was highest during the first 3 years after the index patient initiated treatment and remained higher than the estimated community incidence throughout the duration of follow-up (figure 2B).

**Figure 3:**
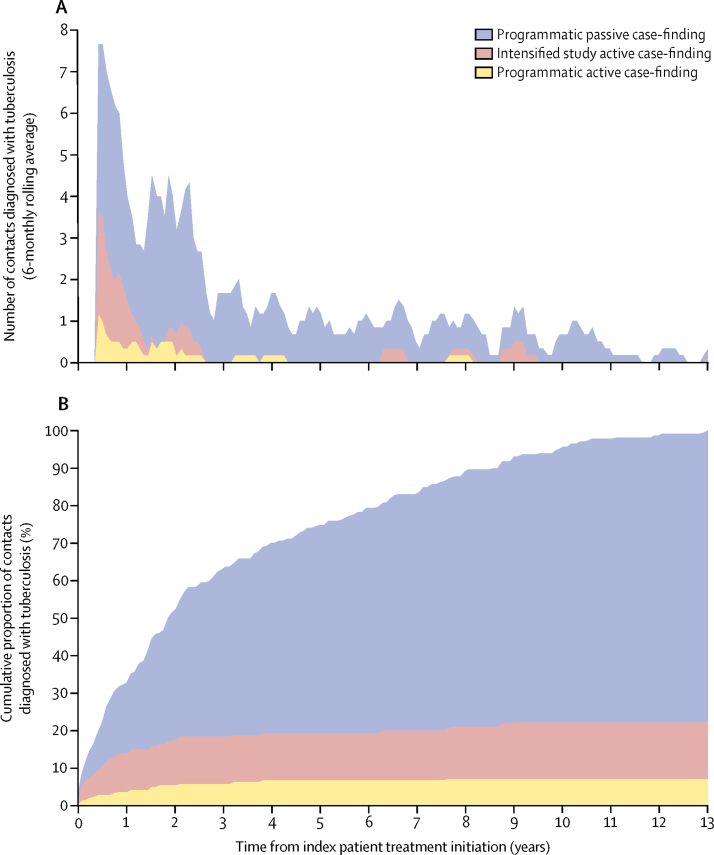
6-monthly rolling average number and cumulative proportion of contacts diagnosed with tuberculosis, by tuberculosis ascertainment strategy

Of 232 contacts diagnosed with tuberculosis, 53 (23%) were diagnosed through all active case-finding (figure 1): 17 (7%) contacts were diagnosed by programmatic active case-finding and 36 (16%) by intensified study active case-finding. The remaining 179 (77%) contacts were diagnosed through programmatic passive case-finding: 158 (88%) were diagnosed by MINSA-run health posts and hospitals and 21 (12% ) by health institutions outside of MINSA. 196 (84%) of 232 diagnoses were confirmed by checking official records. During the first 6 months after the index patient initiated treatment, 51 (22%) of 232 contacts were diagnosed with tuberculosis. Of these, 23 (45%) were diagnosed through all active case-finding and 28 (55%) were diagnosed through programmatic passive case-finding. Figure 3 shows 6-monthly rolling average and cumulative proportions of contacts with tuberculosis, by ascertainment strategy. The number of tuberculosis tests done during intensified study active case-finding is in the appendix.

**Figure 4:**
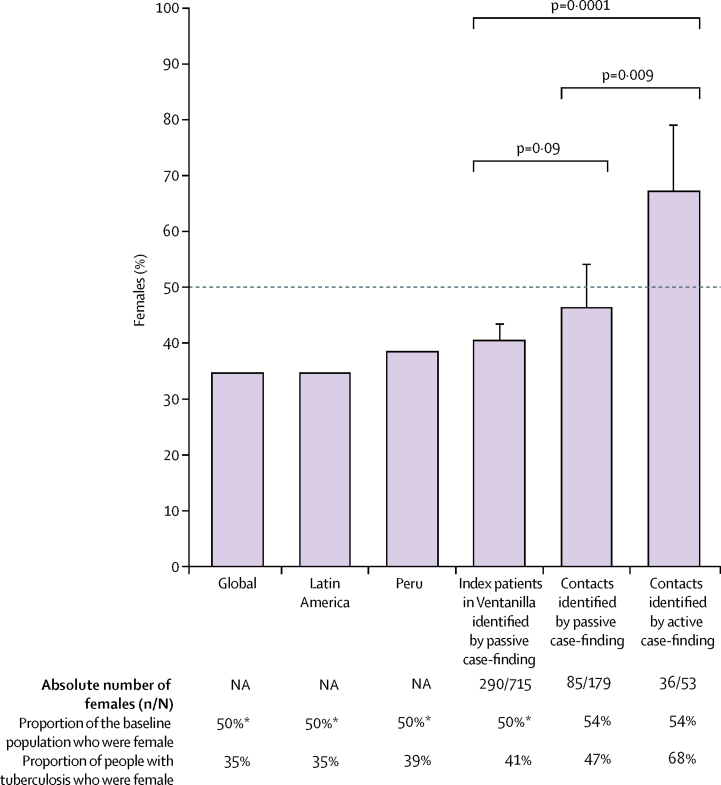
Proportion of people with tuberculosis who were female, by tuberculosis ascertainment strategy Global and Latin American data were derived from the estimated tuberculosis incidence among people aged 15 years or older, reported by WHO.^2^ Peruvian data were derived from routinely available data.^7^ p values indicate two-sample proportion tests. Dotted line at 50% acts as a visual guide to show differences between groups. NA=not available. *Estimate.

Contacts diagnosed through all active case-finding were more likely to be female than were index patients (36 [68%] of 53 *vs* 290 [41%] of 715; p=0·0001; figure 4). 12 (71%) of 17 contacts diagnosed through programmatic active case-finding and 24 (67%) of 36 contacts diagnosed through intensified study active case-finding were female. 85 (47%) of 179 contacts diagnosed through programmatic passive case-finding were female. Contacts diagnosed through all active case-finding were significantly more likely to be female than were contacts diagnosed through programmatic passive case-finding (p=0·009).

**Figure 5:**
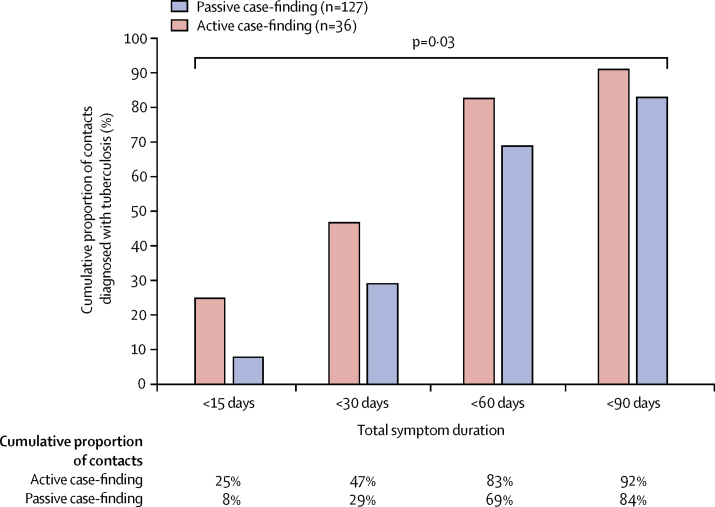
Total symptom duration before diagnosis among contacts with tuberculosis with data available, by tuberculosis ascertainment strategy p value calculated with the Mann-Whitney U test and compares total symptom duration between the two tuberculosis ascertainment strategies.

Overall, contacts diagnosed through all active case-finding had a significantly shorter symptom duration before diagnosis than did contacts diagnosed through passive case-finding (p=0·03; figure 5). The median symptom duration for contacts with data available (n=163) was 30 days (IQR 30–45) for those diagnosed through programmatic active case-finding, 22 days (10–40) for those diagnosed through intensified study active case-finding, and 32 days (20–60) for those diagnosed through programmatic passive case-finding. Of 36 contacts diagnosed through all active case-finding, 17 (47%) had symptoms for less than 30 days compared with 37 (29%) of 127 contacts diagnosed through programmatic passive case-finding. Nine (25%) of 36 contacts diagnosed through all active case-finding had symptoms for less than 15 days compared with ten (8%) of 127 contacts diagnosed through programmatic passive case-finding.

**Figure 6:**
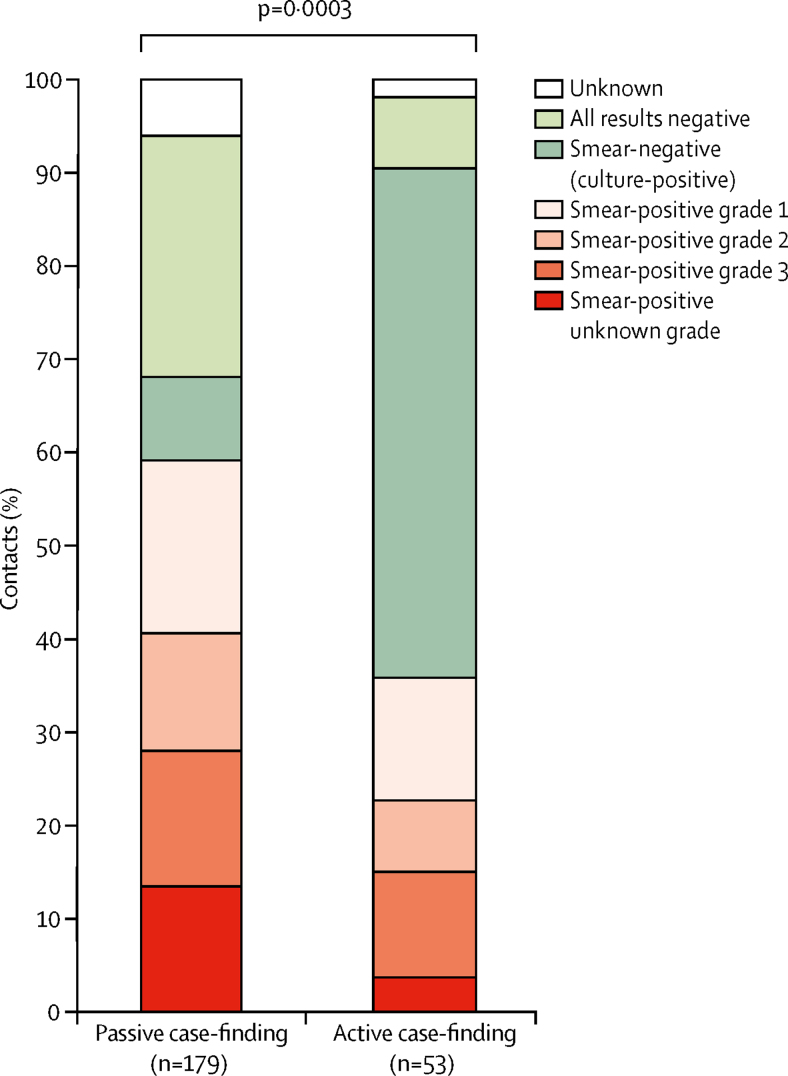
Laboratory results among contacts diagnosed with tuberculosis, by tuberculosis ascertainment strategy p value indicates a two-sample proportion test comparing the proportion of contacts who are smear-negative between the two tuberculosis ascertainment strategies.

Figure 6 shows the distribution of laboratory results for contacts. Overall, 33 (62%) of 53 contacts diagnosed through all active case-finding had sputum smear-negative tuberculosis compared with 62 (35%) of 179 contacts diagnosed through programmatic passive case-finding (p=0·0003). This difference remained significant when considering only contacts with a sputum smear result available, and only contacts who had laboratory-confirmed tuberculosis (appendix). Of 36 contacts diagnosed through intensified study active case-finding, 29 (81%) had sputum smear-negative (culture-positive) tuberculosis. The proportion of contacts who had successful tuberculosis treatment did not differ between contacts diagnosed through all active case-finding and those diagnosed through programmatic passive case-finding (38 [72%] of 53 *vs* 131 [73%] of 179; p=0·8).

The proportion of households in which the head of the household had not completed secondary education did not differ between contacts diagnosed through all active case-finding and those diagnosed through passive case-finding (40 [75%] of 53 *vs* 123 [69%] of 179; p=0·3). Household income did not differ between contacts diagnosed through all active case-finding compared with those diagnosed through passive case-finding (median income US$153 [IQR 122–248] *vs* US$153 [92–248]; p=0·1).

## Discussion

Our prospective cohort study of 2666 contacts aged 15 years or older provides important evidence to support and optimise the early implementation of active case-finding among contacts of patients with tuberculosis in countries of low and middle income. Active case-finding detected a substantial tuberculosis burden among this group, accounting for nearly half of all cases diagnosed in the first 6 months after index patient diagnosis. Intensified study active case-finding (repeated household visits with culture testing) detected more tuberculosis cases than did programmatic active case-finding (relying on contacts to visit health posts for sputum smear microscopy). Compared with passive case-finding, active case-finding was equitable and detected tuberculosis earlier and when contacts were less likely to have highly infectious, sputum smear-positive tuberculosis. Furthermore, in this setting, active case-finding detected tuberculosis in a higher proportion of women than men, providing evidence to support our hypothesis that higher tuberculosis case notifications in men in Peru could reflect a health-care disparity, with passive case-finding failing to adequately detect tuberculosis in women. Despite these findings, passive case-finding detected most of the tuberculosis case burden among contacts, showing the importance of improving both access to, and quality of, passive case-finding for both men and women.

We found a substantial burden of tuberculosis through active case-finding, extending evidence from a cluster-randomised trial that showed the effectiveness of active case-finding among contacts using repeated chest radiography and sputum culture testing for increasing tuberculosis case detection.^17^ We cannot ascertain how often active case-finding accelerated detection of tuberculosis that would have later been diagnosed through passive case-finding nor how active case-finding detected cases that would never have been diagnosed through passive case-finding—eg, because they would have resolved, died, or remained chronically undetected. However, intensified study active case-finding mainly occurred after programmatic active case-finding and detected more cases than did programmatic active case-finding, thus adding considerably to the overall diagnostic yield. Early diagnosis and initiation of appropriate treatment rapidly renders patients with tuberculosis non-infectious, averting further transmission.^18^ Concordantly, mathematical models suggest that active case-finding and treatment of subclinical tuberculosis should have a greater effect towards tuberculosis elimination than would increasing passive case-finding.^19^ Our data support these projections because we found that contacts diagnosed through active case-finding had a shorter symptom duration before diagnosis and, on diagnosis, were more likely to have sputum smear-negative tuberculosis. Taken together, our results suggest that additional case-finding efforts—using more sensitive and rapid diagnostics (potentially including molecular tests) and more relaxed symptomatic criteria for tuberculosis testing among people with known recent tuberculosis exposure—are likely to be vital to the early detection and treatment of tuberculosis in high-burden settings.

Our results suggest that some of the observed sex differences in tuberculosis burden in Peru could be attributable to disparities in access to care under programmatic passive case-finding, and not just differences between the sexes in exposure and susceptibility to tuberculosis. The globally observed predominance of tuberculosis among men has been attributed to increased exposure^20^ or amplified susceptibility because of factors including larger lung volumes, sex hormones, and higher hepcidin levels.^21^, ^22^ In our study, variation of tuberculosis exposure by sex was minimised by studying household contacts of patients with infectious tuberculosis. In this population, we found that women were as likely as men to develop tuberculosis. In other settings, high HIV prevalence is driving an increased tuberculosis burden among women,^23^ but this situation is unlikely in our setting where population HIV prevalence is low (approximately 0·2% of women aged 15–49 years) and only four contacts were known to have HIV.^7^, ^24^ Other explanations for globally higher tuberculosis case notifications among men are that men might have better access to health care or provide better quality sputum for tuberculosis diagnosis.^25^ We found that both intensified study and programmatic active case-finding diagnosed tuberculosis in significantly more women than men, showing a large burden of undetected tuberculosis among women that was overcome by active case-finding. Interpretation of this finding is complex because slightly more contacts were women, and women who might have had more intense exposures to predominantly male index patients, but it is supported by a previous study of contacts in Peru in which women were also significantly more likely to be diagnosed by active case-finding than men.^26^

Peruvian society is known for being particularly patriarchal, reflected by our previous work showing longer diagnostic delay among women,^10^ and places greater community emphasis on men's health, with women's health considered to be of lower priority.^11^ Our results suggest that instructed sputum collection in the privacy of the contact's own home, and perseverance with repeated opportunities for testing, overcame this sex bias of prioritisation of men's health and provided better access for women than did passive case-finding.^15^ This result is important because it shows the potential of active case-finding interventions to provide health-care equity for hard-to-reach underserved groups. These results could also be partly accounted for by men being less likely to access active case-finding if they were less often present during household visits, a hypothesis supported by global data indicating that men are less likely to participate in prevalence surveys.^6^ We do not believe this possibility to be the case in our study, because if men were not present during household visits, another household member was able to complete the screening interview on their behalf and sputum pots were left to facilitate sputum collection at the contact's convenience. Overall, these complexities highlight the importance of further research to investigate equitable strategies to optimise access to tuberculosis testing in both active case-finding and passive case-finding strategies for members of both sexes.

A further novel finding of this study is that passive case-finding accounted for most of the overall tuberculosis burden among contacts. This could in part be because passive case-finding continued for a median of 10 years whereas active case-finding was principally implemented during the first 6 months and then only once every 4 years. However, even during the first 6 months, passive case-finding diagnosed more tuberculosis than did active case-finding. This finding is somewhat surprising and could partly be accounted for by a spillover effect if active case-finding made contacts more aware of tuberculosis symptoms and signs and how to access free tuberculosis testing. Thus, even if a diagnosis was missed by active case-finding or tuberculosis developed afterwards, active case-finding activities might have directly increased subsequent uptake of passive case-finding, highlighting the potential of education as a core component of any active case-finding strategy to increase access to passive case-finding.^27^ It is worth further noting that visiting households at 4-yearly intervals to offer rescreening only detected a few additional cases. In summary, these findings support the idea that active case-finding might need to be done more frequently among contacts, particularly during the first 3 years after exposure, when incidence is highest, to detect more cases at an earlier stage.

Finally, our results characterising the tuberculosis burden among contacts extend those presented in a systematic review and show an overall tuberculosis risk of approximately 5% during the first 2 years after exposure.^28^ We showed that, in this setting, the incidence of tuberculosis among contacts is highest in the first 3 years after exposure and remains higher than the background incidence for at least 10 years, longer than previously shown and indicative of the urgent need to expand the use of preventive treatment for this population, which accounts for many millions of people globally. This persistent risk could be accounted for by a combination of late reactivation of latent tuberculosis infection, reinfection due to ongoing community transmission, and clustering of socioeconomic risk factors within tuberculosis-affected households.

A strength of our study is the comprehensive follow-up to ascertain tuberculosis diagnoses both within the study area at participating MINSA-run health posts using treatment registers and outside of the study area by asking about tuberculosis episodes at other health posts, private clinics, and employer-insured health facilities. Our study also has limitations. Intensified study active case-finding did not include chest radiography, thus, it was unable to diagnose tuberculosis in people without symptoms who had radiological signs suggestive of tuberculosis. Although we reported treatment success proportions that are consistent with previous research in this setting,^27^ we were unable to identify people who were diagnosed with tuberculosis through passive case-finding but who never started treatment, because these people are rarely recorded in national treatment registers. Furthermore, much larger studies are needed to investigate the effect of active case-finding on long-term tuberculosis treatment outcomes, including recurrence and disability. Because active case-finding among contacts aims to diagnose all cases of tuberculosis irrespective of the primary source of infection, we did not use molecular techniques to identify tuberculosis strains and confirm transmission from index patients to contacts because it would not affect our conclusions. Finally, we did not gather data for costs associated with the intensified study active case-finding described. However, active case-finding has been shown to be both feasible and cost-effective in various settings^29^ and, integrated with preventive treatment for contacts, is likely to have an important effect on tuberculosis incidence, making it worth further investment.^30^

Despite widespread implementation in high-income countries, active case-finding among contacts is infrequently implemented and accessed in countries of low and middle income. Our study shows the important contribution active case-finding can make to diagnosing tuberculosis among contacts and suggests that more intensified active case-finding, including using more sensitive diagnostics, has the potential to find more tuberculosis cases. Active case-finding was equitable, diagnosed tuberculosis at an earlier and usually less infectious stage, and showed a high burden of undetected tuberculosis among women, reversing apparent sex differences in tuberculosis notifications in this setting.

## References

[1] Yuen CM, Amanullah F, Dharmadhikari A (2015). Turning off the tap: stopping tuberculosis transmission through active case-finding and prompt effective treatment. Lancet.

[2] WHO (2018). Global tuberculosis report 2018.

[3] WHO (2018). Latent tuberculosis infection: updated and consolidated guidelines for programmatic management.

[4] Saunders MJ, Wingfield T, Tovar MA (2017). A score to predict and stratify risk of tuberculosis in adult contacts of tuberculosis index cases: a prospective derivation and external validation cohort study. Lancet Infect Dis.

[5] Saunders MJ, Datta S (2016). Contact investigation: a priority for tuberculosis control programs. Am J Respir Crit Care Med.

[6] Horton KC, MacPherson P, Houben RMGJ, White RG, Corbett EL (2016). Sex differences in tuberculosis burden and notifications in low- and middle-income countries: a systematic review and meta-analysis. PLoS Med.

[7] Alarcon V, Alarcon E, Figueroa C, Mendoza-Ticona A (2017). Tuberculosis en el Perú: situación epidemiológica, avances y desafíos para su control. Rev Peru Med Exp Salud Publica.

[8] Romero-Sandoval N, Flores-Carrera O, Sanchez-Perez H, Sanchez-Perez I, Mateo M (2007). Pulmonary tuberculosis in an idigenous community in the mountains of Ecuador. Int J Tuberc Lung Dis.

[9] Basta PC, Coimbra CEA, Escobar AL, Santos RV, Alves LCC, de Souza Fonseca L (2006). Survey for tuberculosis in an indigenous population of Amazonia: the Suruí of Rondônia, Brazil. Trans R Soc Trop Med Hyg.

[10] Bonadonna LV, Saunders MJ, Guio H, Zegarra RO, Evans CA (2018). Socioeconomic and behavioral factors associated with tuberculosis diagnostic delay in Lima, Peru. Am J Trop Med Hyg.

[11] Onifade DA, Bayer AM, Montoya R (2010). Gender-related factors influencing tuberculosis control in shantytowns: a qualitative study. BMC Public Health.

[12] Ministerio de Salud (2001). Actualización de la doctrina: normas y procedimientos para el control de la tuberuclosis en el Perú.

[13] Moore DAJ, Evans CAW, Gilman RH (2006). Microscopic-observation drug-susceptibility assay for the diagnosis of TB. N Engl J Med.

[14] Saunders MJ, Tovar MA, Zevallos K (2016). Can micronutrient supplementation prevent TB in vulnerable household contacts? A randomised controlled trial. Int J Tuberc Lung Dis.

[15] Datta S, Shah L, Gilman RH, Evans CA (2017). Comparison of sputum collection methods for tuberculosis diagnosis: a systematic review and pairwise and network meta-analysis. Lancet Glob Health.

[16] Toit K, Mitchell S, Balabanova Y (2012). The Colour Test for drug susceptibility testing of *Mycobacterium tuberculosis* strains. Int J Tuberc Lung Dis.

[17] Fox GJ, Nhung NV, Sy DN (2018). Household-contact investigation for detection of tuberculosis in Vietnam. N Engl J Med.

[18] Dharmadhikari AS, Mphahlele M, Venter K (2014). Rapid impact of effective treatment on transmission of multidrug-resistant tuberculosis. Int J Tuberc Lung Dis.

[19] Dowdy DW, Basu S, Andrews JR (2013). Is passive diagnosis enough? The impact of subclinical disease on diagnostic strategies for tuberculosis. Am J Respir Crit Care Med.

[20] Dodd PJ, Looker C, Plumb ID (2016). Age- and sex-specific social contact patterns and incidence of *Mycobacterium tuberculosis* infection. Am J Epidemiol.

[21] Nhamoyebonde S, Leslie A (2014). Biological differences between the sexes and susceptibility to tuberculosis. J Infect Dis.

[22] Yates TA, Atkinon SH (2017). Ironing out sex differences in tuberculosis prevalence. Int J Tuberc Lung Dis.

[23] Perumal R, Naidoo K, Padayatchi N (2018). TB epidemiology: where are the young women? Know your tuberculosis epidemic, know your response. BMC Public Health.

[24] UNAIDS Country: Peru. http://www.unaids.org/en/regionscountries/countries/peru.

[25] Khan MS, Khine TM, Hutchison C (2016). Are current case-finding methods underdiagnosing tuberculosis among women in Myanmar? An analysis of operational data from Yangon and the nationwide prevalence survey. BMC Infect Dis.

[26] Becerra MC, Pachao-Torreblanca IF, Bayona J (2005). Expanding tuberculosis case detection by screening household contacts. Public Health Rep.

[27] Wingfield T, Tovar MA, Huff D (2017). A randomized controlled study of socioeconomic support to enhance tuberculosis prevention and treatment, Peru. Bull World Health Organ.

[28] Fox GJ, Barry SE, Britton WJ, Marks GB (2013). Contact investigation for tuberculosis: a systematic review and meta-analysis. Eur Respir J.

[29] Azman AS, Golub JE, Dowdy DW (2014). How much is tuberculosis screening worth? Estimating the value of active case finding for tuberculosis in South Africa, China, and India. BMC Med.

[30] Kasaie P, Andrews JR, Kelton WD, Dowdy DW (2014). Timing of tuberculosis transmission and the impact of household contact tracing: an agent-based simulation model. Am J Respir Crit Care Med.

