# Supplementary material for: Active and passive case-finding in tuberculosis-affected households in Peru: a 10-year prospective cohort study
Source: Lancet Infect Dis. 2019 May;19(5):519–28. doi: 10.1016/S1473-3099(18)30753-9 (PMC6483977; doi:10.1016/S1473-3099(18)30753-9)

THE LANCET Infectious Diseases

Supplementary webappendix

This webappendix formed part of the original submission and has been peer reviewed. We post it as supplied by the authors.

Supplement to: Saunders MJ, Tovar MA, Collier D, et al. Active and passive case-finding in tuberculosis-affected households in Peru: a 10-year prospective cohort study. *Lancet Infect Dis* 2019; published online March 22. http://dx.doi.org/10.1016/S1473-3099(18)30753-9.

Appendix Table 1: Summary of numbers of contacts screened by intensified study ACF.

	Testing only symptomatic contacts			Testing all contacts
	During the first six months after recruitment	During the second six months after recruitment	During years 2-10 before the final household visit	At the final household visit (2015-2016)
**Number of contacts eligible for intensified study ACF**	2,666	2,666	2,666	2,666
**Number of TB tests performed**	643	83	268	770

Appendix figure 1a: Cumulative risk of tuberculosis among contacts stratified by sex (n=2666).

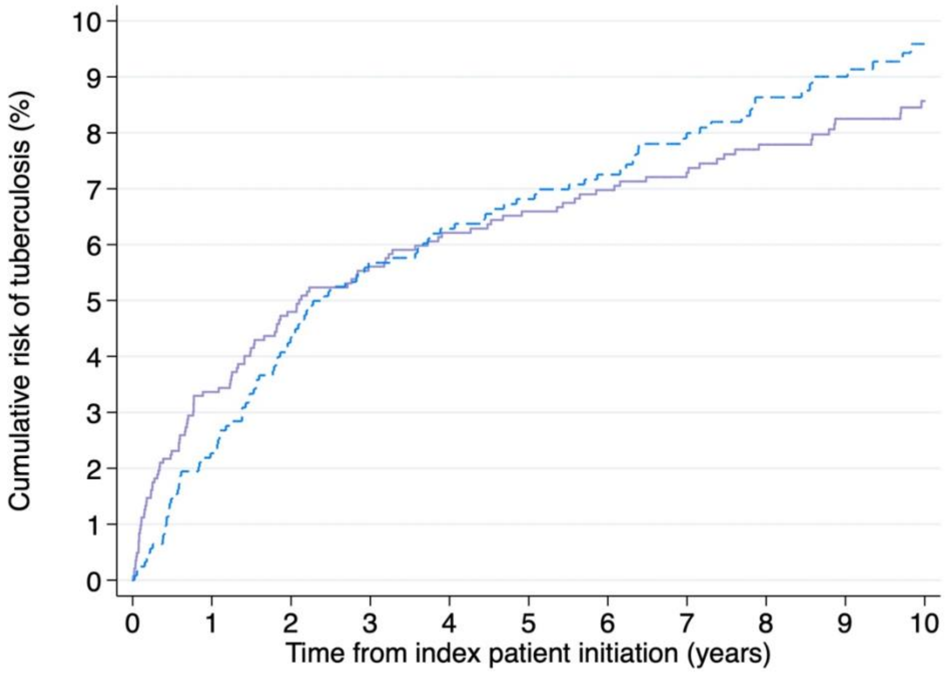

	0	1	2	3	4	5	6	7	8	9	10
**Male contacts (n=1,237)**											
**Number of contacts (tuberculosis diagnoses)**	1237 (28)	1202 (25)	1151 (16)	1097 (7)	1067 (6)	1058 (5)	1042 (8)	948 (6)	812 (3)	693 (4)	512
**Cumulative risk (%) (95% CI)**		2.3 (1.6-3.3)	4.3 (3.3-5.6)	5.7 (4.5-7.1)	6.3 (5.1-7.8)	6.8 (5.5-8.4)	7.3 (5.9-8.9)	8.0 (6.6-10)	8.6 (7.2-10)	9.0 (7.5-11)	10 (8.0-12)
**Female contacts (n=1,429)**											
**Number of contacts (tuberculosis diagnoses)**	1429 (48)	1371 (20)	1317 (11)	1261 (8)	1230 (5)	1220 (5)	1205 (4)	1152 (6)	1059 (5)	975 (3)	795
**Cumulative risk (%) (95% CI)**		3.4 (2.6-4.4)	4.8 (3.8-6.0)	5.6 (4.5-6.9)	6.2 (5.1-7.6)	6.6 (5.4-8.0)	7.0 (5.8-8.5)	7.3 (6.0-8.8)	7.8 (6.5-9.3)	8.3 (6.9-10)	8.6 (7.2-10)

Appendix figure 1b: Incidence of tuberculosis among contacts (n=2666) stratified by sex. Error bars represent 95% confidence intervals.

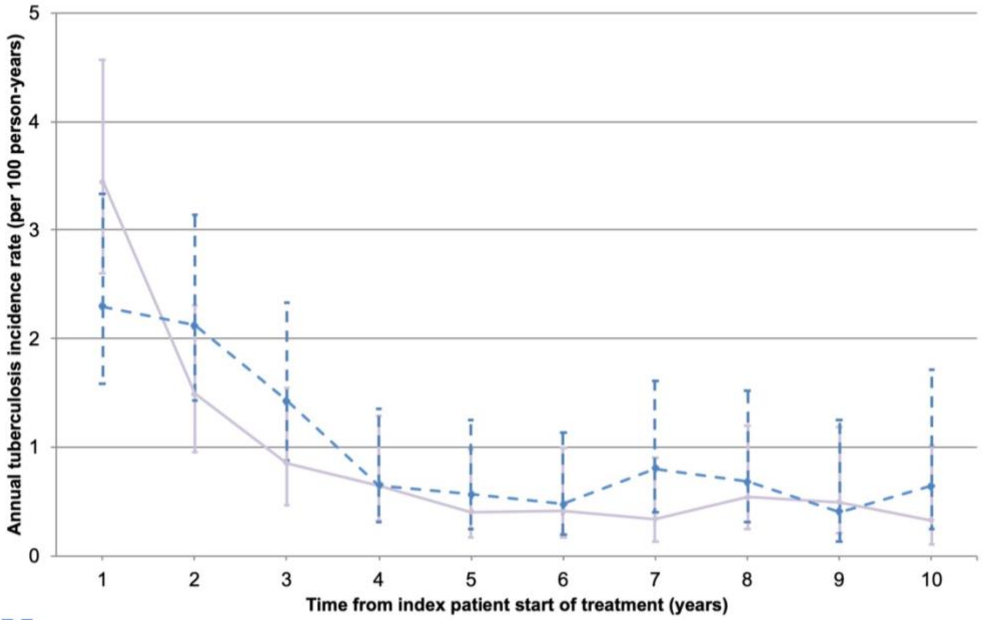

	1	2	3	4	5	6	7	8	9	10
**Male contacts (n=1,237)**										
**Person-years follow up**	1218	1177	1121	1081	1062	1053	993	878	744	620
**Number of tuberculosis cases**	28	25	16	7	6	5	8	6	3	4
**Tuberculosis incidence**	2.3	2.1	1.4	0.65	0.56	0.47	0.81	0.68	0.40	0.65
**Female contacts (n=1,429)**										
**Person-years follow up**	1393	1342	1287	1244	1224	1216	1181	1108	1014	917
**Number of tuberculosis cases**	48	20	11	8	5	5	4	6	5	3
**Tuberculosis incidence**	3.4	1.5	0.85	0.64	0.41	0.41	0.34	0.54	0.49	0.33

Appendix figure 2a: Sputum smear grade results among contacts with data available for smear result (n=181) according to tuberculosis ascertainment strategy. The numbers within the bars indicate percentages. The p value indicates a two-sample proportion test comparing the proportion smear negative between the two ascertainment strategies.

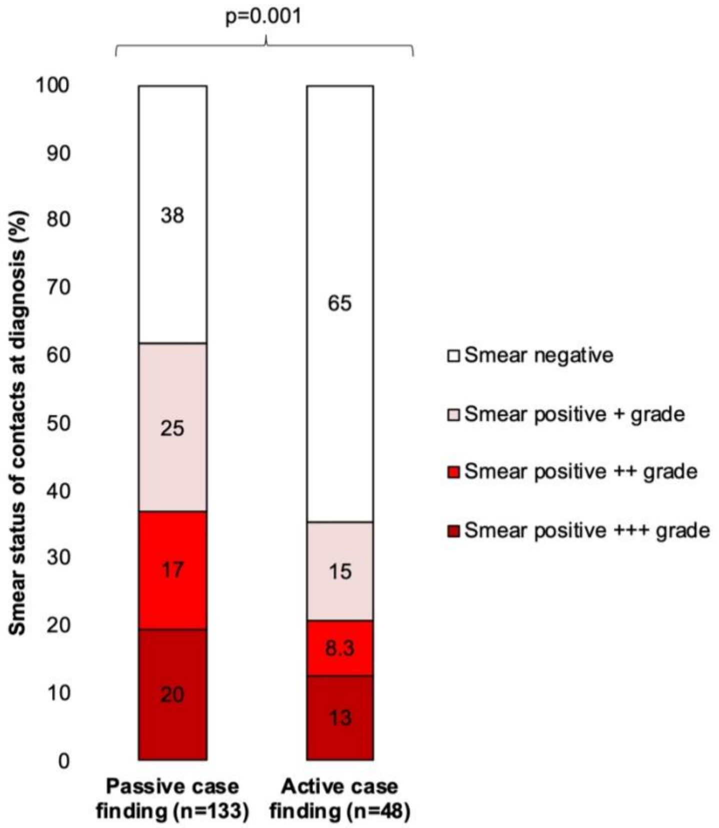

Appendix figure 2b: Sputum smear grade and culture results among contacts with laboratory confirmed tuberculosis (n=170) according to tuberculosis ascertainment strategy. The numbers within the bars indicate percentages. The p value indicates a two-sample proportion test comparing the proportion smear negative, culture positive between the two ascertainment strategies.

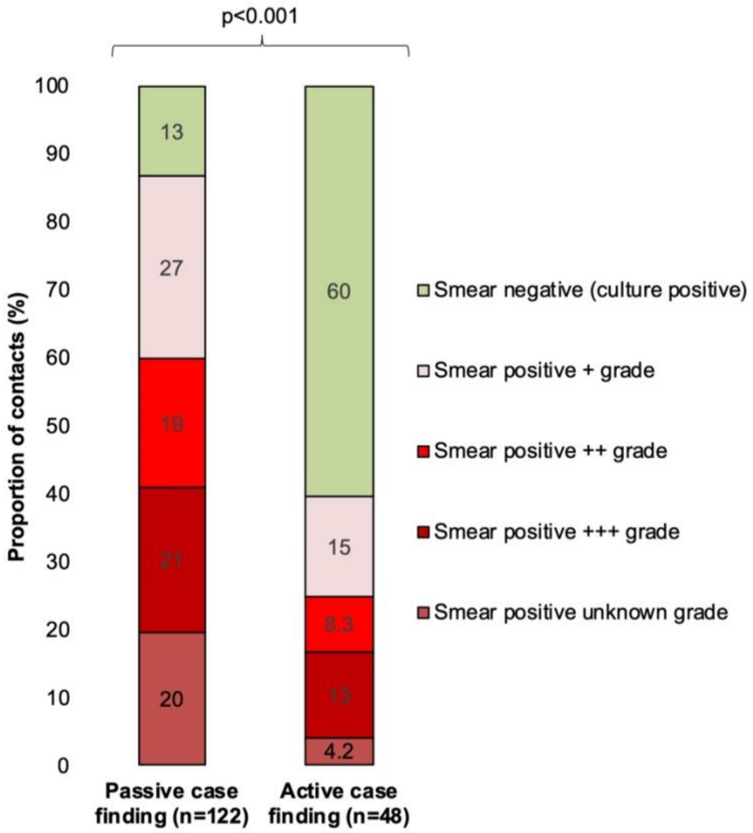

Supplement: Supplementary appendix [file mmc1.pdf]
